# Development of a real-world, therapeutic drug monitoring–informed model to predict teicoplanin daily dose in pediatric intensive care unit patients with bacterial infections

**DOI:** 10.1007/s11096-026-02124-0

**Published:** 2026-04-17

**Authors:** Fusang Wang, Mei Zhang, Suiwen Ye, Jianan Yan, Xuechun Li, Jinyuan Zhang, Xiaoxia Yu, Ying Wang, Ze Yu, Fei Gao, Junyan Wu

**Affiliations:** 1 Department of Pharmacy, Sun Yat-Sen Memorial Hospital, Sun Yat-Sen University, Guangzhou, 510120 China; 2 Phase I Clinical Research Center, Sun Yat-Sen Memorial Hospital, Sun Yat-Sen University, Guangzhou, 510120 China; 3 Beijing Medicinovo Technology Co., Ltd., Beijing, 100163 China

**Keywords:** Teicoplanin, Pediatric intensive care, Therapeutic drug monitoring, Machine learning, TabNet, Individualized dosing, Clinical decision support

## Abstract

**Introduction:**

Teicoplanin is commonly used to treat Gram-positive bacterial infections in the intensive care unit (ICU). However, evidence to support individualized therapeutic drug monitoring (TDM)-guided daily dosing of teicoplanin in pediatric ICU patients remains limited despite substantial interpatient variability in pharmacokinetics and clinical response.

**Aim:**

To develop and validate a real-world TDM-informed machine learning model to predict physician-adjusted teicoplanin daily dose in pediatric ICU patients, with the goal of supporting individualized dosing decisions in clinical pharmacy practice.

**Method:**

Clinical and TDM data from pediatric ICU patients receiving teicoplanin at the Sun Yat-sen Memorial Hospital of Sun Yat-sen University between June 2020 and June 2023 were retrospectively collected. The outcome variable was the daily teicoplanin dose administered during routine TDM-guided clinical care. After univariate screening and sequential forward selection, the dataset was divided into training and test sets (8:2). Missing values were imputed using the random forest approach. Nine machine learning and deep learning algorithms, including gradient boosting, XGBoost, LightGBM, and TabNet, were developed and evaluated using tenfold cross-validation, with model performance assessed using the coefficient of determination (R^2^), root mean square error (RMSE), and mean absolute error (MAE).

**Results:**

A total of 257 pediatric ICU patients (595 teicoplanin dosing records) were included in the study. Weight, age, height, teicoplanin trough concentration (TDM), glucose, creatine kinase isoenzyme-MB, total protein, concomitant imipenem and meropenem use, and upper respiratory infection were identified as key predictors. Among the nine models, the TabNet algorithm demonstrated the best performance on the test set (R^2^ = 0.82, RMSE = 53.96 mg/day, MAE = 39.93 mg/day). The proportion of predictions within ± 30% of the observed daily dose was 81.51%.

**Conclusion:**

This real-world TDM-informed TabNet model shows strong performance in predicting the daily dose of clinician-adjusted teicoplanin in pediatric ICU patients. The model may serve as a clinical decision-support tool for pharmacists and physicians to assist individualized teicoplanin dosing within routine TDM workflows, potentially improving dosing consistency, and supporting safe and effective antimicrobial therapy.

**Supplementary Information:**

The online version contains supplementary material available at 10.1007/s11096-026-02124-0.

## Impact statements

- This study provides a machine learning-based tool to complement and optimize therapeutic drug monitoring-guided teicoplanin dosing in pediatric ICU patients, which may inform clinical pharmacists in TDM-guided dosing decisions.
- The model may assist pharmacists in personalizing and quantifying physician-adjusted daily doses based on real-world complex clinical factors, improving dosing consistency, and reducing the risk of under- or over-dosing that is prone to occur in empirical dose adjustment with single TDM data.
- Integration of the model into routine clinical workflows could standardize the TDM-guided dose adjustment process and enhance individualized antimicrobial therapy, filling the gap of pragmatic and quantitative dosing aids for teicoplanin in the vulnerable pediatric ICU population with physiological heterogeneity and multiple comorbidities.

## Introduction

Teicoplanin is frequently used to treat Gram-positive bacterial infections and is among the most common pathogens encountered in intensive care unit (ICU) [1, 2]. Due to the substantial interindividual variability in teicoplanin pharmacokinetics [3], current clinical guidelines recommend routine therapeutic drug monitoring (TDM) to support individualized dosing and optimize treatment outcomes [4].

Teicoplanin is also widely used in pediatric patients. However, drug disposition in children is highly variable because of age-dependent physiological changes related to growth and maturation, which can markedly influence the pharmacokinetic (PK) properties of many drugs [5]. The elimination half-life of teicoplanin in pediatric patients is comparatively long, approximately 58 h [6]. Evidence suggests that fixed weight-based dosing regimens (6–10 mg/kg/day) may be insufficient to achieve therapeutic exposure in certain pediatric subgroups. For example, standard dosing may result in sub-therapeutic teicoplanin concentrations in children aged 1.0–5.9 years [7]. Suboptimal teicoplanin exposure has been associated with an increased risk of treatment failure, while excessive exposure may increase the risk of adverse events, including nephrotoxicity and hepatotoxicity [8–10]. Importantly, most adverse reactions associated with teicoplanin appear to be dose dependent [11]. Therefore, effective teicoplanin therapy in pediatric ICU patients requires careful balancing of efficacy and safety through individualized dosing strategies, and there is a clear need for decision-support tools that can assist physicians and pharmacists in optimizing teicoplanin daily doses and reducing the risks of underdosing or overdosing.

Machine learning (ML) enables computers to identify complex patterns within high-dimensional datasets and generate predictive models that can be iteratively validated and optimized [12, 13]. In recent years, ML-based approaches have been increasingly applied in biomedical and clinical pharmacy research, including in disease diagnosis, health monitoring, and individualized pharmacotherapy [14]. In the context of teicoplanin therapy, ML models have been used to predict plasma teicoplanin concentrations based on key clinical factors, such as age, weight, and creatinine clearance (CL) [15]. More broadly, ML and deep learning techniques have shown promise in supporting personalized drug dosing, as demonstrated by previously reported models for vancomycin dosing using extreme gradient boosting (XGBoost) and warfarin maintenance dose prediction using light gradient boosting machine (LightGBM) [16, 17]. Despite these advances, evidence regarding the application of machine-learning models to support teicoplanin daily dose determination, particularly in pediatric ICU patients under routine TDM-guided care, remains limited.

Therefore, optimizing the teicoplanin daily dose in pediatric ICU patients remains a significant clinical challenge. Most existing studies have focused on pharmacokinetic modeling or concentration prediction, whereas tools designed to support real-world clinician-adjusted dosing decisions are scarce. Leveraging routinely collected clinical and TDM data through ML may offer a pragmatic approach to support individualized teicoplanin dosing in this vulnerable population.

## Aim

This study aimed to develop and validate a real-world, TDM-informed machine learning model to predict physician-adjusted teicoplanin daily dose in pediatric ICU patients.

## Method

### Study design and participants

This retrospective study included pediatric patients who received teicoplanin therapy and were admitted to the ICU of Sun Yat-sen Memorial Hospital of Sun Yat-sen University between June 2020 and June 2023.

The inclusion criteria were as follows: (1) age ≤ 14 years, (2) receipt of teicoplanin therapy for at least 2–3 days to allow attainment of steady-state concentrations, and (3) availability of therapeutic drug monitoring (TDM) data for teicoplanin trough concentration. The exclusion criteria were as follows: (1) teicoplanin concentrations below the lower limit of quantification and (2) cases with substantial missing or incomplete clinical data.

According to package insert, the recommended teicoplanin dosing regimen for pediatric patients aged 2 months to 12 years consists of a loading dose of 10 mg/kg every 12 h for three doses, followed by a maintenance dose of 6–10 mg/kg once daily. Teicoplanin trough concentrations were routinely measured from 72 h onwards after treatment initiation [18, 19]. This sampling window was chosen to ensure that samples were collected closer to the steady-state concentrations, which is critical for accurate TDM given teicoplanin’s long half-life (approximately 58 h in children). TDM samples were consistently drawn immediately prior to the next scheduled dose, in accordance with standard trough concentration measurement protocols. To ground our model in real-world practice, we describe the teicoplanin TDM workflow used at our center, consistent with standard TDM principles described in recent reviews [20, 21]. In routine clinical practice at our center, teicoplanin dosing decisions were guided by the institutional TDM service using a predefined dosing protocol. When trough concentrations were outside the target range or when clinical status changed, clinicians adjusted the teicoplanin daily dose using predefined dosing increments within approved dose bands. The final daily dose for each patient was then determined by the attending pharmacist in collaboration with the physician, based on a comprehensive clinical assessment that integrated the trough concentration with relevant patient-specific factors such as organ function, infection characteristics, and concomitant therapies. This standardised dose-adjustment process defines the real-world context that our model aims to emulate. In this study, the observed teicoplanin daily dose administered in clinical practice was defined as the outcome variable for model development. Thus, the predicted outcome reflects the physician-adjusted daily dose under routine TDM-guided care, chosen to capture real-world dosing decisions and support dosing consistency, rather than a theoretical “optimal” dose derived solely from PK or population pharmacokinetic (PPK) modeling.

### Data collection

The dataset comprised teicoplanin-related variables, demographic characteristics (age, age stratification, sex, weight, height), comorbidities (hypertension, diabetes, hyperlipidemia, coronary heart disease, stroke, congenital heart disease, chronic obstructive pulmonary disease [COPD], hepatic insufficiency, chronic kidney disease [CKD], renal insufficiency, acute myeloid leukemia [AML], hypoproteinemia, hematologic malignancy, sepsis, organ malignancy, autoimmune disease, myocardial damage, liver abscess), infection sites (lung infection, upper respiratory infection, bone infection, bloodstream infection], intra-abdominal infection), concomitant medications (amphotericin B, imipenem, flexbumin, furosemide, cyclosporine, ceftriaxone, meropenem, supportive therapies (peritoneal dialysis, hemodialysis, extracorporeal membrane oxygenation [ECMO], tracheotomy, blood transfusion, mechanical ventilation), and laboratory parameters (glucose [GLU], creatine kinase isoenzyme-MB [CK-MB], and total protein [TP]).

### Data preprocessing

To improve model performance and comparability across variables, continuous variables were standardized using z-score normalization and categorical variables were transformed using one-hot encoding. Variables with a missing rate greater than 50% were excluded from further analyses. Additionally, highly imbalanced binary variables (defined as a positive sample proportion of < 10%) were removed to reduce model instability. To assess the potential impact of preprocessing steps (such as feature selection, imputation) on model results, a dedicated sensitivity analysis was implemented. All preprocessing procedures were learned independently on the training data within each fold and then applied to the corresponding test data. This approach aimed to evaluate any potential bias in the conclusions that might arise from the initial preprocessing strategy.

### Feature selection and missing data imputation

Univariate analyses were performed to explore the associations between candidate variables and the teicoplanin daily dose. Variables with a *P* value < 0.05 were retained for subsequent feature selection. Missing values among the retained variables were imputed using a random-forest-based imputation approach. Sequential forward selection (SFS) was then applied to identify the optimal subset of predictors for model construction.

### Model development and validation

Supplemental Figure S1 shows the overall modeling workflow. The dataset was randomly divided into a training set and a test set in an 8:2 ratio. The training set was used for hyperparameter optimization via grid search and for model development using tenfold cross-validation, whereas the test set was reserved for the final performance evaluation. Regarding the repeated dosing records, each record was treated as an independent observation in the present analysis. This approach was justified by the fact that each record corresponds to a unique, TDM-guided dose adjustment event, reflecting dynamic changes in the patient’s clinical status, physiological parameters, concomitant medications, and infection-related characteristics at the time of TDM assessment-all of which are core determinants of the final clinician-adjusted teicoplanin daily dose.

Nine ML and deep learning algorithms were developed for teicoplanin daily dose prediction: gradient boosting machine (GBM), XGBoost, LightGBM, categorical boosting (CatBoost), adaptive boosting (AdaBoost), random forest (RF), support vector machine (SVM), multilayer perceptron (MLP), and TabNet. To enhance reproducibility and provide a concise, self-contained description, the most critical TabNet hyperparameters are summarized here. The TabNet model was configured with the following key settings: number of decision steps n_steps = 4, attention dimension n_a = 8, output dimension n_d = 8, batch_size = 64, virtual_batch_size = 32, momentum = 0.2, gamma = 1.0, max_epochs = 500, and early_stopping_patience = 50. The complete configuration and tuning details for all models are provided in Supplementary Tables S1 and S2.

The model performance was evaluated using the coefficient of determination (R^2^), root mean square error (RMSE), and mean absolute error (MAE), calculated as follows:

$$\mathrm{R}2=1-\frac{MSE\left(\widehat{y},y\right)}{\mathit{Var}\left(y\right)}$$

$$\mathrm{RMSE}=\sqrt{\frac{1}{\mathrm{n}}{\sum }_{\mathrm{i}=1}^{\mathrm{n}}{\left({\mathrm{y}}_{\mathrm{i}}-{\widehat{\mathrm{y}}}_{\mathrm{i}}\right)}^{2}}$$

$$\mathrm{MAE}=\frac{1}{n}\sum_{i=1}^{n}|{\widehat{y}}_{i}-{y}_{i}|$$

where ${\widehat{y}}_{i}$ denotes the predicted teicoplanin daily dose, $i,{y_i}$ denotes the observed daily dose, and *n* represents the number of observations. $MSE\mathrm{(}\widehat{y}\mathrm{,}y\mathrm{)=}\frac{1}{n}\sum_{i= \mathrm{1} }^{n}\mathrm{(}{y}_{i}-{\widehat{y}}_{i}{)}^{2}$ is the mean squared error, and $Var(y)$ is the empirical variance of the observed daily doses. Higher R2 values and lower RMSE and MAE values indicated better predictive performance.

The algorithm that demonstrated the best overall performance for the test set was selected as the final prediction model.

### Model interpretation

To enhance interpretability, feature importance scores were generated for the optimal model. The Shapley Additive exPlanations (SHAP) method and TabNet mask analysis were applied to quantify and visualize the contribution of individual features to the model predictions [22]. MASK matrices were used to illustrate the feature importance across decision steps at the individual patient level.

### Statistical analysis

Baseline characteristics were evaluated for associations with daily teicoplanin dose. The Kolmogorov–Smirnov test was used to assess data normality. For normally distributed data, independent t-tests were applied to categorical variables, and Pearson’s correlation analysis was applied to continuous variables. For non-normally distributed data, Mann–Whitney U tests and Spearman’s correlation analysis were used. Statistical analyses were performed using SPSS version 25.0, and machine-learning analyses were conducted using Python version 3.7.

### Ethics approval

The study was approved by the Ethics Committee of the Sun Yat-sen Memorial Hospital, Sun Yat-sen University (No. SYSKY-2023–815-02).

## Results

### Patient selection and baseline characteristics

**Fig. 1:**
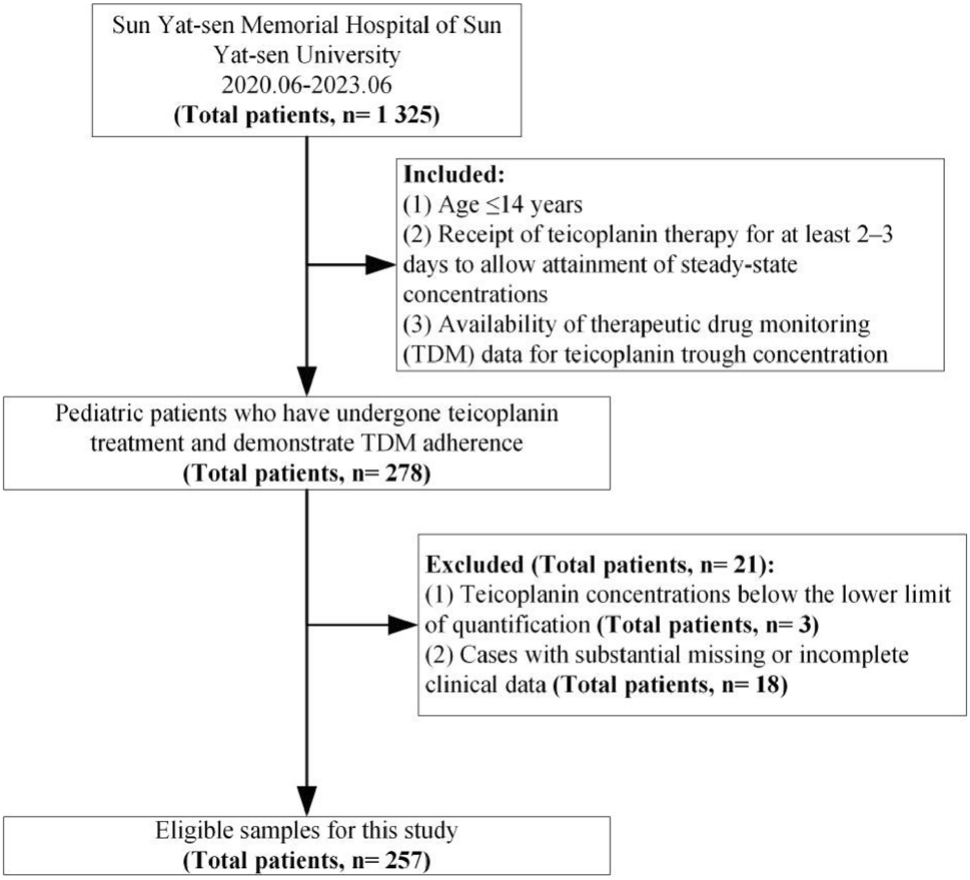
Flow diagram of patient inclusion and exclusion

Of 1,325 pediatric ICU admissions, 257 patients contributing 595 teicoplanin dosing records were included in the final analysis after applying the inclusion and exclusion criteria (Fig. 1)**.** The baseline demographic and clinical characteristics are summarized in Table 1. The median age of the patients was 6.00 years (interquartile range [IQR], 3.00–9.00), with a median weight of 19.65 kg (IQR, 13.77–26.00) and a median height of 115.00 cm (IQR, 98.00–132.00). Patients aged ≥ 1 and ≤ 14 years accounted for 95.80% of the cohort and 62.86% were male.

**Table 1:** Baseline demographic and clinical characteristics of pediatric ICU patients receiving teicoplanin

Category	Variable	Median (IQR) | n (%)	Miss rate
Teicoplanin information	Daily dose, mg	200.00 (150.00–300.00)	0.00%
Teicoplanin trough concentration	TDM, mg/L	8.53 (5.12–12.71)	0.00%
Demographic information	Age, years	6.00 (3.00–9.00)	0.00%
	Age stratification		0.00%
	1 ~ 14 years	570 (95.80%)	
	≤ 1 year	25 (4.20%)	
	Gender		0.00%
	Male	374 (62.86%)	
	Female	221 (37.14%)	
	Height, cm	115.00 (98.00–132.00)	6.55%
	Weight, kg	19.65 (13.77–26.00)	0.17%
Combined disease	Hypertension	17 (2.86%)	0.00%
	Diabetes	17 (2.86%)	0.00%
	Hyperlipidemia	11 (1.85%)	0.00%
	Coronary heart disease	0 (0.0%)	0.00%
	Stroke	0 (0.0%)	0.00%
	Congenital heart disease	1 (0.17%)	0.00%
	COPD	0 (0.0%)	0.00%
	Hepatic insufficiency	6 (1.01%)	0.00%
	CKD	5 (0.84%)	0.00%
	Renal insufficiency	19 (3.19%)	0.00%
	AML	184 (30.92%)	0.00%
	Hypoproteinemia	153 (25.71%)	0.00%
	Hematologic malignancy	328 (55.13%)	0.00%
	Sepsis	114 (19.16%)	0.00%
	Organ malignancy	21 (3.53%)	0.00%
	Autoimmune disease	7 (1.18%)	0.00%
	Myocardial damage	2 (0.34%)	0.00%
	Liver abscess	8 (1.34%)	0.00%
Infection site	Lung infection	414 (69.58%)	0.00%
	Upper respiratory infection	179 (30.08%)	0.00%
	Bone infection	0 (0.0%)	0.00%
	Bloodstream infection	1 (0.17%)	0.00%
	Intra-abdominal infection	8 (1.34%)	0.00%
Concomitant medication	Amphotericin B	68 (11.43%)	0.00%
	Imipenem	144 (24.20%)	0.00%
	Flexbumin	180 (30.25%)	0.00%
	Furosemide	385 (64.71%)	0.00%
	Ceftriaxone	4 (0.67%)	0.00%
	Cyclosporine	138 (23.19%)	0.00%
	Meropenem	246 (41.34%)	0.00%
Supportive therapy	Peritoneal dialysis	0 (0.0%)	0.00%
	Hemodialysis	0 (0.0%)	0.00%
	ECMO	0 (0.0%)	0.00%
	Tracheotomy	1 (0.17%)	0.00%
	Blood transfusion	401 (67.39%)	0.00%
	Mechanical ventilation	23 (3.87%)	0.00%
Laboratory test	ALT, U/L	20.00 (11.00–44.50)	23.53%
	AST, U/L	26.00 (17.50–45.50)	22.18%
	TBIL, μmol/L	7.60 (5.20–12.90)	24.37%
	DBIL, μmol/L	3.30 (2.10–5.40)	28.57%
	IBIL, μmol/L	3.80 (2.50–5.90)	30.08%
	CR, μmol/L	27.00 (21.00–38.00)	25.88%
	UA, μmol/L	172.00 (124.00–235.00)	33.28%
	UREA, mmol/L	4.50 (3.20–6.60)	27.56%
	LDH, U/L	223.00 (167.00–357.00)	32.27%
	ALB, g/L	37.40 (33.92–40.58)	25.04%
	CRP, mg/L	16.70 (4.93–64.21)	2.18%
	G, g/L	25.85 (21.40–30.98)	25.71%
	CK, IU/L	25.00 (15.00–40.50)	34.29%
	CK-MB, IU/L	15.00 (10.00–24.00)	38.82%
	ProCT, ng/mL	0.26 (0.14–0.62)	41.68%
	EOS, 10^9^/L	0.00 (0.00–0.02)	23.03%
	EOS%, %	0.00 (0.00–0.88)	13.61%
	BASO, 10^9^/L	0.00 (0.00–0.01)	23.03%
	BASO%, %	0.00 (0.00–0.30)	13.95%
	MONO, 10^9^/L	0.25 (0.05–0.58)	23.03%
	MONO%, %	14.10 (6.30–25.50)	13.78%
	MCV, fl	84.40 (80.60–89.28)	1.51%
	NEU, 10^9^/L	0.64 (0.08–2.12)	23.03%
	NEU%, %	44.40 (19.12–65.88)	23.03%
	HBDH, U/L	178.50 (136.00–261.75)	38.82%
	PLT, 10^9^/L	49.00 (24.25–105.00)	10.92%
	MPV, fl	10.80 (10.00–11.80)	20.67%
	PCT, %	0.80 (0.40–1.32)	30.08%
	PDW, fl	11.80 (10.20–13.80)	30.08%
	P-LCR, %	31.40 (23.88–37.73)	20.67%
	TT, s	17.40 (16.40–18.60)	49.58%
	TCO2, mmol/L	21.70 (19.80–24.00)	32.61%
	Fbg C, s	0.64 (0.08–2.12)	23.03%
	GLU, mmol/L	5.08 (4.41–5.95)	17.98%
	PTA%, %	99.50 (85.20–114.65)	49.75%
	PT, s	11.10 (10.40–12.30)	49.75%
	APTT, s	29.30 (26.10–34.00)	49.58%
	HGB, g/L	78.00 (70.00–90.00)	1.85%
	RBC, 10^9^/L	2.74 (2.38–3.11)	10.92%
	HCT, L/L	0.23 (0.20–0.27)	10.92%
	RDW-CV, %	14.60 (12.90–17.50)	11.76%
	WBC, 10^9^/L	1.12 (0.20–3.50)	10.92%
	LYM, 10^9^/L	0.34 (0.13–0.81)	23.03%
	LYM%	27.50 (11.80–54.20)	13.78%
	MCHC, g/L	338.00 (327.00–349.00)	1.51%
	RDW-SD, %	45.10 (39.80–52.90)	2.35%
	MCH, pg	28.70 (27.30–30.10)	1.51%
	TP, g/L	63.40 (57.60–69.90)	25.21%
	Na, mmol/L	137.00 (135.00–139.00)	6.39%
	Mg, mmol/L	0.79 (0.69–0.88)	14.45%
	Cl, mmol/L	102.60 (99.70–105.20)	7.06%
	P, mmol/L	1.45 (1.17–1.71)	14.79%
	K, mmol/L	4.02 (3.66–4.40)	6.39%
	Ca, mmol/L	2.28 (2.18–2.37)	8.07%

TDM, therapeutic drug monitoring; COPD, chronic obstructive pulmonary disease; CKD, chronic kidney disease; AML, acute myeloid leukemia; ECMO, extracorporeal membrane oxygenation; ALT, alanine transaminase; AST, aspartate transaminase; TBIL, total bilirubin; DBIL, direct bilirubin; IBIL, indirect bilirubin; CR, creatinine; UA, uric acid; LDH, lactic dehydrogenase; ALB, albumin; CRP, C-reactive protein; G, globulin; CK, creatine kinase; CK-MB, creatine kinase isoenzyme-MB; ProCT, procalcitonin; EOS, eosinophils; EOS%, eosinophils ratio; BASO, basophil count; BASO%, basophil ratio; MONO, monocytes; MONO%, monocytes ratio; MCV, mean corpuscular volume; NEU, total neutrophil count; NEU%, total neutrophil count ratio; HBDH, hydroxybutyrate dehydrogenase; PLT, platelet count; MPV, mean platelet volume; PCT, plateletcrit; PDW, platelet distribution width; P-LCR, platelet larger cell ratio; TT, thrombin time; TCO2, total carbon dioxide; Fbg C, fibrinogen coagulative time; GLU, glucose; PTA%, prothrombin activity; PT, prothrombin time; APTT, activated partial thromboplastin time; HGB, hemoglobinometry; RBC, red blood count; HCT, hematocrit; RDW-CV, red cell distribution width-coefficient variation; WBC, white blood count; LYM, total lymphocytes; LYM%, total lymphocytes ratio; MCHC, mean corpusular hemoglobin concerntration; RDW-SD, red cell distribution width-standard deviation; MCH, mean corpusular hemoglobin; TP, total protein; Na, -sodium; Mg, magnesium; Cl, chlorine; P, phosphorus; K, potassium; Ca, calcium

All continuous variables are shown as median values and interquartile ranges (IQR) and categorical variables as frequencies and percentages

The median teicoplanin trough concentration was 8.53 mg/L (IQR, 5.12–12.71), and the median teicoplanin daily dose was 200.00 mg/day (IQR, 150.00–300.00). Among the comorbidities, hematologic malignancy was the most prevalent (55.13%), followed by AML (30.92%), hypoproteinemia (25.71%), and sepsis (19.16%). Regarding the infection sites, lung infection (69.58%) and upper respiratory infection (30.08%) were predominant.

Regarding concomitant medications, furosemide (64.71%) was the most frequently prescribed, followed by meropenem (41.34%), flexbumin (30.25%), and imipenem (24.20%). Blood transfusions were the most common supportive therapy (67.39%). Median laboratory values included TP 63.40 g/L (IQR, 57.60–69.90), GLU 5.08 mmol/L (IQR, 4.41–5.95), and CK-MB 15.00 IU/L (IQR, 10.00–24.00).

### Feature selection

Initially, 96 candidate variables were extracted from a clinical database. Eight variables with missing rates > 30% and 36 highly imbalanced variables were excluded, leaving 52 variables for univariate analysis. Among these, 30 variables were significantly associated with teicoplanin daily dose (*P* < 0.05) (Table 2).

**Table 2:** Univariate analysis of factors associated with teicoplanin daily dose

Category	*P*-value	Category	*P*-value
Weight	< 0.001	age	< 0.001
Height	< 0.001	NEU	0.659
TDM	< 0.001	Na	0.587
LYM%	0.050	IBIL	0.144
TT	0.120	MPV	0.369
RDW-SD	0.415	LYM	0.121
TCO2	0.011	MONO%	0.173
RBC	< 0.001	UREA	0.758
HGB	0.011	DBIL	0.904
Ca	0.351	PCT	< 0.001
P	0.603	PLT	< 0.001
NEU%	0.046	PT	0.042
RDW-CV	0.255	TBIL	0.967
HCT	0.016	K	< 0.001
CK	< 0.001	EOS%	0.602
CRP	0.122	MCH	< 0.001
P-LCR	0.433	APTT	0.638
GLU	0.008	G	< 0.001
Fbg C	0.002	CK_MB	< 0.001
ALB	0.862	TP	0.003
AST	< 0.001	ProCT	0.381
BASO	0.210	MCHC	0.057
ALT	0.041	gender	0.007
UA	0.415	AML	0.889
MCV	< 0.001	hypoproteinemia	0.330
HBDH	0.024	hematologic malignancy	0.012
BASO%	0.127	sepsis	0.791
WBC	0.622	lung infection	0.522
MONO	0.646	upper respiratory infection	< 0.001
LDH	0.026	Amphotericin B	0.346
PDW	0.054	Imipenem	< 0.001
Mg	< 0.001	Flexbumin	0.550
Cl	0.010	Furosemide	0.184
PTA%	0.080	Cyclosporine	0.181
EOS	0.930	meropenem	< 0.001
CR	< 0.001	blood transfusion	0.205

TDM, therapeutic drug monitoring; ALT, alanine transaminase; AST, aspartate transaminase; TBIL, total bilirubin; DBIL, direct bilirubin; IBIL, indirect bilirubin; CR, creatinine; UA, uric acid; LDH, lactic dehydrogenase; ALB, albumin; CRP, C-reactive protein; G, globulin; CK, creatine kinase; CK-MB, creatine kinase isoenzyme-MB; ProCT, procalcitonin; EOS, eosinophils; EOS%, eosinophils ratio; BASO, basophil count; BASO%, basophil ratio; MONO, monocytes; MONO%, monocytes ratio; MCV, mean corpuscular volume; NEU, total neutrophil count; NEU%, total neutrophil count ratio; HBDH, hydroxybutyrate dehydrogenase; PLT, platelet count; MPV, mean platelet volume; PCT, plateletcrit; PDW, platelet distribution width; P-LCR, platelet larger cell ratio; TT, thrombin time; TCO2, total carbon dioxide; Fbg C, fibrinogen coagulative time; GLU, glucose; PTA%, prothrombin activity; PT, prothrombin time; APTT, activated partial thromboplastin time; HGB, hemoglobinometry; RBC, red blood count; HCT, hematocrit; RDW-CV, red cell distribution width-coefficient variation; WBC, white blood count; LYM, total lymphocytes; LYM%, total lymphocytes ratio; MCHC, mean corpusular hemoglobin concerntration; RDW-SD, red cell distribution width-standard deviation; MCH, mean corpusular hemoglobin; TP, total protein; Na, sodium; Mg, magnesium; Cl, chlorine; P, phosphorus; K, potassium; Ca, calcium

These 30 variables were subsequently screened using XGBoost-based SFS to identify the optimal predictor combination. Model performance (R^2^) increased progressively as variables were added, reaching a maximum of 0.5604 when 10 variables were included, after which performance declined with additional variables (Supplemental Figure S2).

To balance model parsimony and predictive accuracy, the following ten variables were selected for the final model development: weight, age, height, teicoplanin trough concentration (TDM), GLU, CK-MB, TP, concomitant imipenem use, concomitant meropenem use, and upper respiratory infection.

### Model performance

**Fig. 2:**
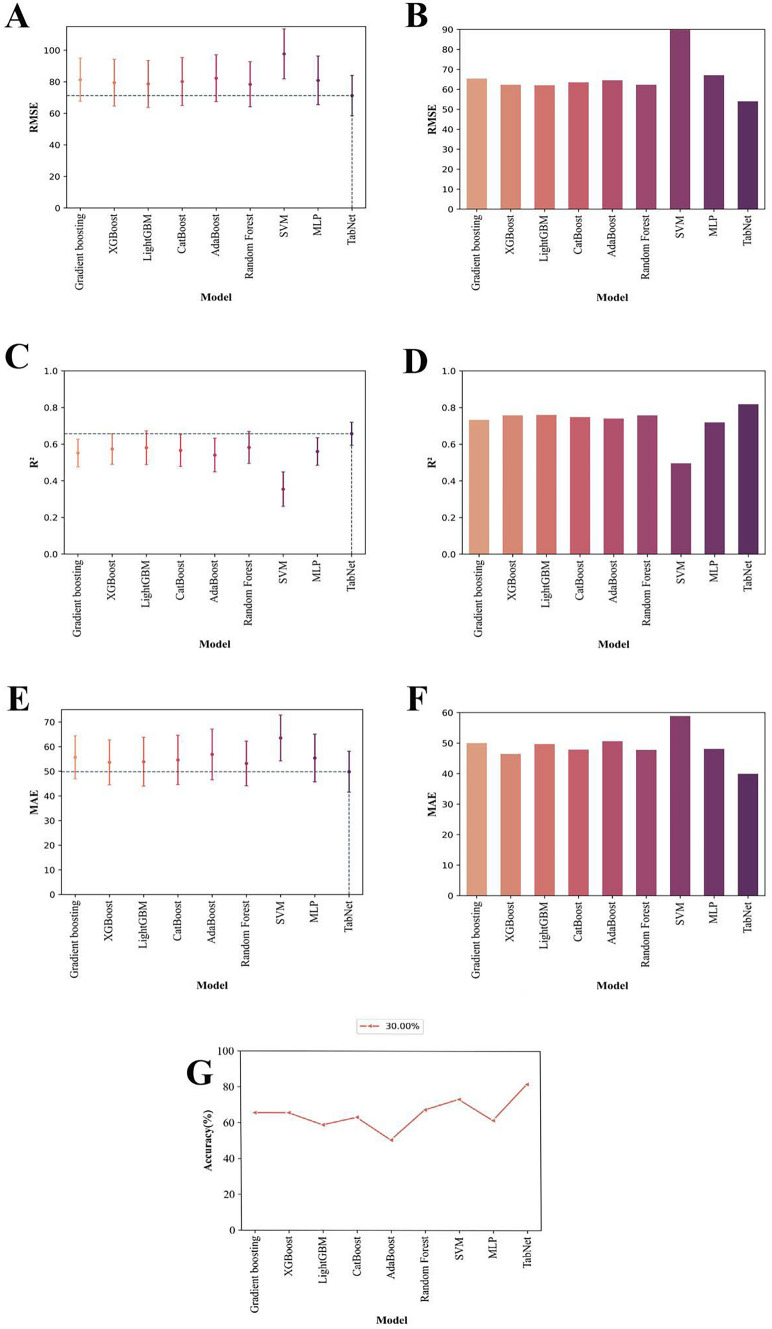
Comparison of predictive performance across machine learning models for teicoplanin daily dose prediction. Panels **A** and **B** show the root-mean-square error (RMSE; mean ± standard deviation) for the training and test sets, respectively. Panels **C** and **D** show the coefficients of determination (R^2^; mean ± standard deviation) for the training and test sets, respectively. Panels **E** and **F** show the mean absolute error (MAE; mean ± standard deviation) for the training and test sets, respectively. Panel **G** shows the proportion of predictions within ± 30% of the observed daily teicoplanin dose in the test set. *Abbreviations* R^2^, coefficient of determination; RMSE, root mean square error; MAE, mean absolute error

Using the selected variables, nine ML and deep-learning models were constructed to predict the teicoplanin daily dose. Ten-fold cross-validation demonstrated that the TabNet model consistently outperformed other algorithms (Fig. 2; Supplemental Table S3).

Across cross-validation folds, the TabNet model achieved a mean RMSE of 71.23 ± 12.17 mg/day, a mean R^2^ of 0.66 ± 0.06, and a mean MAE of 49.82 ± 7.87 mg/day. When evaluated on the independent test set, TabNet achieved an R^2^ of 0.82, RMSE of 53.96 mg/day, and MAE of 39.93 mg/day (Supplemental Table S4).

A dedicated sensitivity analysis showed that TabNet performed consistently, achieving an R^2^ of 0.85 (Supplementary Table S5), with RMSE, MAE, and ± 30% accuracy remaining comparable to the initial findings.

### Model interpretation

A feature importance analysis based on the TabNet model is presented in Table 3 and Supplemental Figure S3. The three most influential predictors were weight (importance score 0.219), concomitant imipenem use (0.204), and height (0.180).

**Table 3:** Relative importance of predictors for teicoplanin daily dose based on the TabNet model

Feature	Importance
Weight	0.219
Imipenem	0.204
Height	0.180
TDM	0.096
Age	0.080
TP	0.070
Meropenem	0.053
Upper respiratory infection	0.053
GLU	0.042
CK-MB	0.002

TDM, therapeutic drug monitoring; TP, total protein; GLU, glucose; CK-MB, creatine kinase isoenzyme-MB

**Fig. 3:**
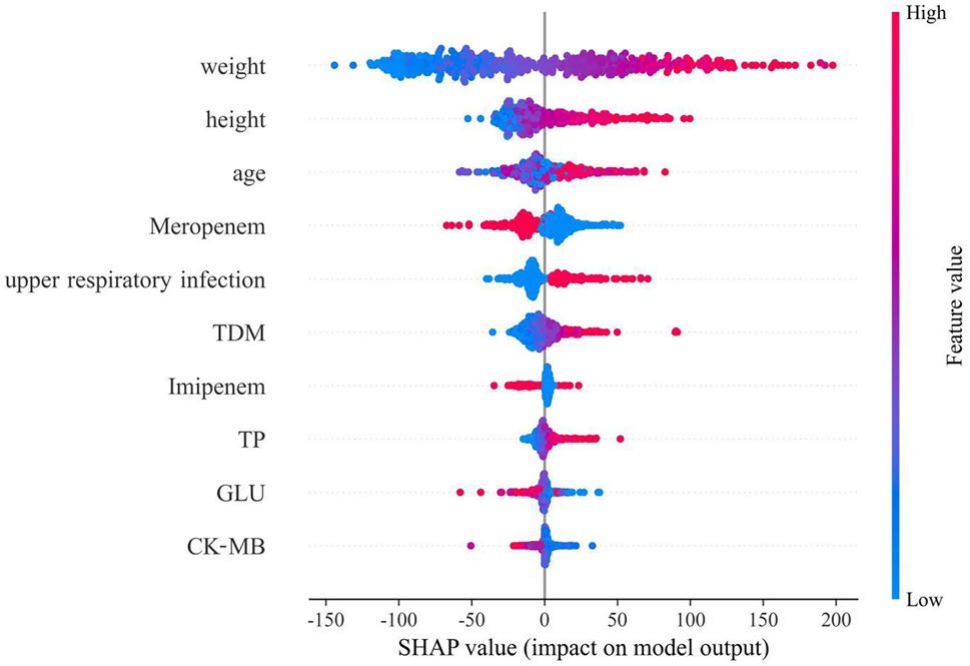
SHAP summary plot showing the contribution of key predictors to teicoplanin daily dose in the TabNet model. Each dot represents an individual observation point. Red indicates higher feature values and blue indicates lower feature values. Positive SHAP values indicate an increase in the predicted teicoplanin daily dose, whereas negative values indicate a decrease. *Abbreviations* TDM, therapeutic drug monitoring; TP, total protein; GLU, glucose; CK-MB, creatine kinase isoenzyme-MB. (Colour figure online)

Statistical testing of the ten selected predictors demonstrated significant associations with teicoplanin daily dose (Supplemental Table S6). SHAP analysis was used to visualize both the direction and magnitude of the associations between predictors and teicoplanin daily dose (Fig. 3). Body weight, height, age, TDM, upper respiratory infection, and TP were positively associated with teicoplanin daily dose, whereas GLU, concomitant meropenem use, and CK-MB were negatively associated. The direction of the effect of imipenem on teicoplanin daily dose was less consistent across patients.

To further examine the feature contributions at the individual level, TabNet mask analysis was performed (Supplemental Figure S4; Table S7). With n_steps set to four, four MASK matrices (119 × 10) were generated for the test set. Weight, height, TDM, concomitant imipenem use, and age consistently demonstrated high importance across decision steps, with body weight emerging as the most influential predictor overall.

Scatter plots and residual analyses comparing predicted and observed teicoplanin daily doses are shown in Supplemental Figure S5, confirming a good model fit and absence of major systematic bias.

### Model application

An online TabNet-based prediction tool was developed to facilitate clinical applications (Supplemental Figure S6). By entering values for the ten selected variables, physicians and pharmacists can obtain an estimated teicoplanin daily dose consistent with real-world TDM-guided dosing practice, thereby supporting individualized clinical decision-making.

## Discussion

### Main findings

This study retrospectively analyzed real-world clinical data to compare the performance of multiple ML and deep learning algorithms in predicting the clinician-adjusted teicoplanin daily dose in pediatric ICU patients with infections. Among the evaluated models, TabNet demonstrated the best overall performance, achieving an R^2^ of 0.82 on the independent test set, indicating strong predictive capability. Rather than replacing TDM or conventional PK/pharmacodynamic (PD) and PPK approaches, the proposed model was designed to serve as a clinical decision-support tool that provides data-driven and quantitative individualized estimates for physician-adjusted daily dose in routine clinical practice, by integrating TDM trough concentrations with ten clinically validated key covariates (such as weight, age). This integration addresses the limitations of traditional PK principle-based dose adjustment that only relies on TDM trough concentrations as a surrogate marker of area under the curve (AUC), thereby filling a critical unmet need in pediatric clinical pharmacies for quantitative dosing tools adapted to the complex clinical setting of pediatric ICU and facilitating more consistent TDM-guided dosing decisions in clinical practice.

### Interpretation

A systematic clinical rationale underpins the selection of the ten key predictors identified in this study for teicoplanin daily dose in pediatric ICU patients, each linked to teicoplanin’s PK, PD, clinical safety considerations, or real-world dosing decision-making in pediatric critical care. Weight, age and height are core demographic predictors, as pediatric teicoplanin disposition is governed by age-dependent physiological maturation and body size-related volume of distribution and CL [7, 23, 24]. TDM is a direct indicator of therapeutic exposure, with TDM-guided dosing being the gold standard for balancing teicoplanin efficacy and safety [5, 11]. TP is critical for teicoplanin PK due to its ~ 90% plasma protein binding; higher TP reduces free drug availability, requiring dose optimization [25]. GLU alters teicoplanin disposition in pediatric ICU-associated hyperglycemic hypoalbuminemia, and metabolic status directly impacts clinician dosing choices [26]. CK-MB marks myocardial/renal injury, with elevated levels prompting cautious dosing to mitigate teicoplanin-induced nephrotoxicity [27]. Concomitant imipenem/meropenem use poses synergistic nephrotoxicity risks, and upper respiratory infection serves as a clinical proxy for infection severity-both core to physician-adjusted dosing [7, 28]. Collectively, these ten variables integrate pediatric-specific PK/PD traits, TDM core principles, clinical safety biomarkers, and real-world prescribing behavior, rendering them the optimal clinical and statistical predictors for teicoplanin daily dose in pediatric ICU patients.

To further clarify the clinical and statistical links between the ten key predictors identified above and teicoplanin dosing, we used univariate analysis and SHAP-based interpretation to elucidate their directional and magnitude effects on teicoplanin daily dose in real-world pediatric ICU practice. Previous pharmacokinetic studies have shown that drug CL increases with age in pediatric patients. Tarral et al. reported higher CL rates in older children than in newborns [23], consistent with the findings of Holford et al. [24]. An age-related increase in CL necessitates higher daily doses to achieve the target therapeutic concentrations, which aligns with the positive association between age and teicoplanin daily dose observed in our SHAP analysis. Similarly, the impact of the total dose per weight on teicoplanin CL was most pronounced during the initial treatment period and followed a concentration-dependent pattern [29]. The strong and consistent influence of weight on the teicoplanin daily dose identified in our model reflects well-established pharmacokinetic principles and is supported by previous studies that demonstrated a linear relationship between weight and CL [30].

Height was an important predictor in our model. Ramos-Martín et al. investigated the influence of renal function on teicoplanin pharmacokinetics using the estimated glomerular filtration rate (eGFR) calculated by the Haycock–Schwarz formula, in which height is a key variable, and observed a linear relationship between eGFR and CL [31]. Our SHAP results suggest that greater height may be associated with increased teicoplanin CL, thereby necessitating higher daily doses, which further supports the biological plausibility of the model.

In addition to demographic factors, several laboratory parameters, infection characteristics, and concomitant medications were associated with teicoplanin daily dose, including TP, CK-MB, GLU, TDM, upper respiratory infection, and concomitant meropenem use. Teicoplanin exhibits extensive plasma protein binding, predominantly to albumin, with approximately 90% of the drug bound and an unbound fraction of 6–12% [25]. The positive association between TP and teicoplanin daily dose observed in our SHAP analysis is consistent with the reduced free drug availability at higher protein levels, potentially requiring higher doses to maintain therapeutic exposure.

Patients with hyperglycemic hypoalbuminemia exhibited lower serum teicoplanin concentrations after a loading dose [26]. In our study, higher GLU levels were negatively associated with the teicoplanin daily dose, suggesting that metabolic status may influence teicoplanin disposition or dosing decisions, although the underlying mechanisms remain unclear and warrant further investigation. CK-MB, primarily a cardiac marker, was identified as a significant negative predictor for teicoplanin daily dose. In critically ill pediatric patients, elevated CK-MB, while indicative of myocardial injury, can also signal broader systemic stress or organ dysfunction, potentially including renal involvement [32]. This is particularly relevant as acute kidney injury (AKI) is prevalent in this vulnerable population and teicoplanin-induced nephrotoxicity is a recognized concern [27, 33]. Although direct evidence linking CK-MB specifically to teicoplanin-related nephrotoxicity in children remains limited, studies have explored the association between cardiac biomarkers and AKI risk in pediatric patients under various physiological stresses, such as during cardiac surgery [34]. Consequently, the observed negative association likely reflects a prudent clinical strategy: clinicians may reduce teicoplanin daily dose in pediatric patients with elevated CK-MB, interpreting it as an indicator of increased fragility and heightened risk for adverse outcomes, including renal injury. This underscores the critical role of clinical judgment in individualized antibiotic dosing..

The importance of TDM in achieving the target teicoplanin concentrations, particularly during early treatment, has been well documented. Dose adjustments based on TDM are frequently required to optimize efficacy and safety [35]. The positive relationship between the TDM values and teicoplanin daily dose observed in our analysis highlights the central role of TDM-guided dose escalation or de-escalation in routine practice, reinforcing the clinical relevance of the proposed model. This reflects real-world dose escalation in response to subtherapeutic concentrations rather than a causal PK relationship. Concomitant imipenem (24.2%) and meropenem (41.3%) use with teicoplanin was common in our cohort. Consistent with literature reporting increased AKI risk with teicoplanin and carbapenems [28], our SHAP analysis showed a notable and consistent negative association between meropenem co-administration and teicoplanin daily dose, whereas the effect of imipenem on teicoplanin daily dose was inconsistent across patients despite its second-highest feature importance score (0.204) among all predictors. This discrepancy may be attributed to two core clinical factors: first, the heterogeneous infection severity in pediatric ICU patients with life-threatening or multidrug-resistant infections [36], where clinicians may increase teicoplanin dose for sufficient antimicrobial efficacy even with imipenem co-administration, while reducing the dose for mild infections to avoid synergistic nephrotoxicity; second, the interindividual variation in clinicians’ risk–benefit assessment for combination antimicrobial therapy [37], with different weights placed on therapeutic efficacy and renal safety in clinical decision-making. In contrast, the higher clinical utilization rate of meropenem has formed a more unified clinical consensus on dose reduction for nephrotoxicity caution. This personalized dosing strategy for carbapenem-teicoplanin combination therapy aligns with established guidelines for drug dosing in renal dysfunction and TDM [38].

Several PPK-based dosing strategies have been proposed to optimize pediatric teicoplanin therapy [30, 39]. PPK modeling, commonly implemented using nonlinear mixed-effects models such as NONMEM [40], provides mechanistic insights into drug disposition, but often requires intensive data collection and may be limited by the availability of clinical outcome data [41, 42]. In contrast, the present ML approach leverages routinely collected real-world clinical and TDM data to model the synergistic effects of multi-dimensional clinical factors and TDM trough concentrations on clinician-adjusted dosing decisions, offering a pragmatic and resource-efficient complementary tool to traditional PK and PPK approaches rather than a simple alternative. This tool fully considers the unique physiological characteristics of pediatric ICU patients (such as age-dependent PK changes) and complex clinical conditions (such as multiple concomitant medications), which are difficult to be systematically incorporated into traditional PK/PPK models in routine clinical practice. While PPK models are based on explicit mathematical assumptions and may be less flexible in accommodating complex interactions [43], ML models can adaptively learn nonlinear relationships from high-dimensional data, enabling individual predictions based on real-world practice patterns [44].

ML has been increasingly applied in precision medicine to support personalized dose adjustment, drug concentration prediction, and adverse event risk assessment [17, 45–47]. In the present study, the TabNet model outperformed the traditional tree-based and neural network algorithms, likely because of its ability to capture nonlinear feature interactions while maintaining interpretability through instance-wise feature selection [48]. TabNet has previously demonstrated a strong performance in real-world individualized dosing models, including those for lapatinib and venlafaxine [49, 50]. By translating the optimized model into an online clinical decision-support tool, this study provides a practical interface through which pharmacists and physicians can integrate model-based dose estimates into routine TDM workflows, potentially improving dosing consistency and supporting safe and effective antimicrobial therapies.

### Implications for practice

Beyond its methodological innovation integrating therapeutic drug monitoring with advanced ML, the TabNet model holds clear clinical translation potential. In the management of severe bacterial infections in pediatric intensive care, it provides robust support for safer, more effective, and efficient individualized teicoplanin dosing. By aligning with real-world clinical decision-making logic and quantifying the impact of multi-dimensional clinical factors on teicoplanin dosing, this tool can facilitate the standardization of TDM-guided dose adjustment processes and personalized pharmacotherapy in pediatric ICU patients. It addresses the long-standing unmet need for pragmatic, rapid, and quantitative dosing aids in vulnerable pediatric populations, where empirical dose adjustment based on single TDM data and clinical experience is still the mainstream in clinical practice.

### Strengths and limitations

This study integrates TDM data for dose prediction, leveraging real-world evidence. The TabNet model offers favorable predictive performance and interpretability, with a supporting online tool to facilitate clinical translation.

This study has several limitations. First, the retrospective, single-center design and exclusion of patients with extensive missing data may limit generalizability, and prospective multicenter studies are needed to validate the findings in a broader pediatric population. Second, although the TabNet model demonstrated strong internal performance, external validation using independent datasets has not yet been performed, and the model robustness in other clinical settings remains uncertain. A multicenter external validation study is currently planned. Third, feature screening and missing-value imputation were conducted prior to dataset splitting, and the model development relied on a single random 8:2 train-test split combined with cross-validation, which may introduce information leakage and optimistic performance estimates. All analyses were exploratory and hypothesis-generating; future work will apply fully nested cross-validation and external validation. Fourth, repeated dosing records from the same patient were treated as independent observations in our analysis, which may introduce within-patient correlation due to shared time-invariant physiological, pharmacokinetic, and clinical management-related similarities across observations from the same individual. This may result in a slight overestimation of model performance in the test set and a marginally optimistic assessment of the mode’s generalizability to external patient cohorts. Future prospective multicenter validation studies will adopt a patient-level split strategy (i.e., assigning all records from a single patient to either the training or test set exclusively) to eliminate within-patient correlation and provide a more conservative and robust assessment of generalizability. Finally, time-dependent clinical factors, including dynamic changes in renal function, were not incorporated, and integrating longitudinal data may further enhance the model performance and clinical applicability.

## Conclusion

Using real-world clinical and therapeutic drug monitoring data, this study developed a machine learning-based model to predict clinician-adjusted teicoplanin daily dose. The TabNet model demonstrated strong predictive performance within routine TDM-guided practice. This approach may complement traditional TDM and PK/PPK-based dose adjustment principles to support pharmacists and physicians in precision individualizing teicoplanin therapy for pediatric ICU patients. By integrating TDM trough concentrations with multi-dimensional clinical covariates, it may improve dosing consistency and support safer and more effective antimicrobial therapy in the complex pediatric ICU settings.

## Supplementary Information

Below is the link to the electronic supplementary material.

Supplementary file1 (DOCX 669 KB)

## Data Availability

The datasets generated and analyzed in the current study are available from the corresponding author upon reasonable request.
