# Supplementary material for: Development of a real-world, therapeutic drug monitoring–informed model to predict teicoplanin daily dose in pediatric intensive care unit patients with bacterial infections
Source: Int J Clin Pharm. 2026 Apr 17;48(4):1462–75. doi: 10.1007/s11096-026-02124-0 (PMC13369723; doi:10.1007/s11096-026-02124-0)
Supplement: Supplementary file 1 — Supplementary file1 (DOCX 669 KB) [file 11096_2026_2124_MOESM1_ESM.docx]

**Development of a Real-World, Therapeutic Drug Monitoring–Informed Model to Predict Teicoplanin Daily Dose in Pediatric Intensive Care Unit Patients with Bacterial Infections**

**Fusang Wang^#1^, Mei Zhang^#1^, Suiwen Ye^#2^, Jianan Yan^3^, Xuechun Li^3^, Jinyuan Zhang^3^, Xiaoxia Yu^1^, Ying Wang^1^, Ze Yu^*3^, Fei Gao^*3^, Junyan Wu^*1,2^**

^1^Department of Pharmacy, Sun Yat-sen Memorial Hospital, Sun Yat-sen University, Guangzhou, 510120, China.

^2^Phase I Clinical Research Center, Sun Yat-sen Memorial Hospital, Sun Yat-sen University, Guangzhou, 510120, China

^3^Beijing Medicinovo Technology Co., Ltd., Beijing, 100163, China

# These authors contributed equally to this work and share first authorship.

* These authors contributed equally to this work.

*Correspondence to:

Junyan Wu

Address: 107 Yanjiang West Road, Guangzhou, 510120, China.

E-mail: [759329969@q](mailto:wujunyan@mail.sysu.edu.cn)q.com

Fei Gao

Address: 17 Yuyuantan South Road, Haidian District, Beijing, 100080, China

E-mail: gaofei9000@163.com

Ze Yu

Address: 17 Yuyuantan South Road, Haidian District, Beijing, 100080, China

E-mail: [15910865863@163.com](mailto:15910865863@163.com)

**Supplementary Tables**

**Table S1.** Cross validation parameters

| Model | Parameters |
| --- | --- |
| Gradient boosting | cv_params = {  'n_estimators': [100, 200, 300, 400, 500, 600],  'learning_rate': [i / 100 for i in range(1, 20)],  'max_depth': [3, 4, 5, 6, 8],  'subsample': [0.5, 0.6, 0.7, 0.8]  } |
| XGBoost | cv_params = {  'n_estimators': [100, 200, 300, 400, 500, 600],  'learning_rate': [i / 100 for i in range(1, 20)],  'max_depth': [3, 4, 5],  'gamma': [i/10.0 for i in range(0, 10)],  'min_child_weight': [0.5, 1, 3, 5, 7],  'reg_alpha': [0.1, 1, 2, 3, 5, 10, 15, 20],  'reg_lambda': [0.1, 1, 2, 3, 5, 8, 10],  'subsample': [0.6, 0.7, 0.8, 0.9, 1],  'colsample_bytree': [0.5, 0.6, 0.7, 0.8, 0.9, 1]  } |
| LightGBM | cv_params = {  'n_estimators': [100, 200, 300, 400, 500, 600, 700],  'learning_rate': [i / 100 for i in range(1, 20)],  'max_depth': [3, 4, 5, 6],  'num_leaves': [7, 15, 31, 62],  'subsample': [0.7, 0.8, 0.9, 1.0],  'colsample_bytree': [0.8, 0.9, 1.0],  'reg_alpha': [0, 1, 2, 3],  'reg_lambda': [0, 1, 5, 6]  } |
| CatBoost | cv_params = {  'iterations': [100, 200, 300, 400, 500],  'learning_rate': [i / 100 for i in range(1, 20)],  'depth': [3, 4, 5],  } |
| AdaBoost | cv_params = {  'n_estimators': [100, 200, 300, 400, 500],  'learning_rate': [x/100 for x in range(1,20)],  'loss':['linear', 'square', 'exponential']  } |
| Random Forest | cv_params = {  'n_estimators': [100, 200, 300, 400, 500, 600],  'max_depth': [3, 4, 5, 6, 7, 8, 9],  'min_samples_split': [3, 4, 5, 6, 7, 8, 9, 10],  'min_samples_leaf': [3, 4, 5, 6, 7, 8, 9, 10],  'max_features': [3, 4, 5, 6, 7, 8, 9, 10, 15],  } |
| SVM | cv_params = {  'C': [0.1, 1, 10],  'epsilon': [0.01, 0.1, 0.2],  'kernel': ['linear', 'rbf', 'poly', 'sigmoid', 'relu']  } |
| MLP | cv_params = {  'hidden_layer_sizes': [(20,),(50,),(100,),(200,)],  'batch_size': [32,64,128,'auto'],  'activation': ['logistic', 'tanh', 'sigmoid', 'identity', 'relu'],  'solver': ['adam','lbfgs', 'sgd'],  'alpha': [0.0001, 0.05],  'learning_rate': ['constant','adaptive'],  'max_iter': [100, 200, 300, 400, 500]  } |
| TabNet | cv_params = {  'max_epochs': [300, 400, 500, 600, 700],  'patience': [5, 10, 20, 50],  'batch_size': [16, 32, 64, 128],  'virtual_batch_size': [16, 32, 64, 128],  'n_d': [8, 16, 32, 64],  'n_a': [8, 16, 32, 64],  'n_step': [3, 4, 5, 6, 7, 8],  'gamma': [1.0, 1.3, 1.4, 1.5],  'momentum': [0.01, 0.02, 0.2, 0.3]  } |

Abbreviations: XGBoost, extreme gradient boosting; LightGBM, light gradient boosting machine; CatBoost, categorical boosting; AdaBoost, adaptive boosting; SVM, support vector machine; MLP, multilayer perceptron.

**Table S2.** Optimal parameters in the ten models

| Model | Optimal parameters |
| --- | --- |
| Gradient boosting | {  'n_estimators': 500,  n_estimators ': 0.15,  'max_depth ': 4,  'subsample': 0.5  } |
| XGBoost | {  'colsample_bytree': 1.0,  'gamma': 0.5,  'learning_rate': 0.17,  'max_depth': 3,  'min_child_weight': 0.5,  'n_estimators': 600,  'reg_alpha': 0.1,  'reg_lambda': 8,  'subsample': 0.5  } |
| LightGBM | {  'colsample_bytree': 1.0,  'learning_rate': 0.01,  'max_depth': 6,  'n_estimators': 500,  'num_leaves': 7,  'reg_alpha': 0.0,  'reg_lambda': 0.0,  'subsample': 1.0,  } |
| CatBoost | {  'iterations': 400,  'learning_rate': 0.02,  'depth': 5,  } |
| AdaBoost | {  'learning_rate': 0.01,  'loss': 'exponential',  'n_estimators': 300,  } |
| Random Forest | {  'bootstrap': True,  'max_depth': 7,  'max_features': 6,  'min_samples_leaf': 5,  'min_samples_split': 3,  'min_weight_fraction_leaf': 0.0,  'n_estimators': 100,  } |
| SVM | {  'C': 10,  'epsilon': 0.01,  'kernel': 'linear',  } |
| MLP | {  'activation': 'logistic',  'alpha': 0.05,  'batch_size': 32,  'hidden_layer_sizes': (20,),  'learning_rate': 'constant',  'max_iter': 200,  'solver': 'lbfgs'  } |
| TabNet | {  'batch_size': 64,  'gamma': 1.0,  'max_epochs': 500,  'momentum': 0.2,  'n_a': 8,  'n_d': 8,  'n_steps': 4,  'patience': 50,  'virtual_batch_size': 32,  } |

Abbreviations: XGBoost, extreme gradient boosting; LightGBM, light gradient boosting machine; CatBoost, categorical boosting; AdaBoost, adaptive boosting; SVM, support vector machine; MLP, multilayer perceptron.

**Table S3.** Results of the 10-fold cross-validation

| Algorithm | RMSE | R² | MAE |
| --- | --- | --- | --- |
| Gradient boosting | 81.30 ± 13.01 | 0.55 ± 0.07 | 55.64 ± 8.31 |
| XGBoost | 79.43 ± 14.10 | 0.57 ± 0.08 | 53.64 ± 8.67 |
| LightGBM | 78.61 ± 14.15 | 0.58 ± 0.09 | 53.88 ± 9.42 |
| CatBoost | 80.12 ± 14.44 | 0.57 ± 0.08 | 54.60 ± 9.50 |
| AdaBoost | 82.23 ± 14.09 | 0.540 ± 0.087 | 56.87± 9.76 |
| Random Forest | 78.39 ± 13.54 | 0.582 ± 0.083 | 53.17 ± 8.62 |
| SVM | 97.68 ± 15.01 | 0.354 ± 0.089 | 63.51± 8.82 |
| MLP | 80.86 ± 14.61 | 0.560 ± 0.071 | 55.37 ± 9.22 |
| TabNet | 71.23 ± 12.17 | 0.656 ± 0.060 | 49.82 ± 7.87 |

Abbreviations: R^2^, coefficient of determination; RMSE, root mean square error; MAE, mean absolute error; XGBoost, extreme gradient boosting; LightGBM, light gradient boosting machine; CatBoost, categorical boosting; AdaBoost, adaptive boosting; SVM, support vector machine; MLP, multilayer perceptron.

**Table S4.** Results of the test set validation

| Algorithm | RMSE | R² | MAE |
| --- | --- | --- | --- |
| Gradient boosting | 65.39 | 0.73 | 49.99 |
| XGBoost | 62.29 | 0.76 | 46.42 |
| LightGBM | 62.02 | 0.76 | 49.69 |
| CatBoost | 63.48 | 0.75 | 47.88 |
| AdaBoost | 64.48 | 0.74 | 50.64 |
| Random Forest | 62.29 | 0.76 | 47.76 |
| SVM | 89.77 | 0.50 | 58.87 |
| MLP | 67.04 | 0.72 | 48.09 |
| TabNet | 53.96 | 0.82 | 39.93 |

Abbreviations: R^2^, coefficient of determination; RMSE, root mean square error; MAE, mean absolute error; XGBoost, extreme gradient boosting; LightGBM, light gradient boosting machine; CatBoost, categorical boosting; AdaBoost, adaptive boosting; SVM, support vector machine; MLP, multilayer perceptron.

**Table S5.** Sensitivity analysis results of the test set validation

| Algorithm | RMSE | R² | MAE | ±30% (%) |
| --- | --- | --- | --- | --- |
| Gradient boosting | 72.49 | 0.67 | 54.10 | 63.03 |
| XGBoost | 65.84 | 0.73 | 50.10 | 69.75 |
| LightGBM | 58.35 | 0.79 | 40.88 | 72.27 |
| CatBoost | 58.50 | 0.79 | 40.83 | 72.27 |
| AdaBoost | 59.75 | 0.78 | 43.75 | 68.07 |
| Random Forest | 57.00 | 0.80 | 41.07 | 76.47 |
| SVM | 75.43 | 0.64 | 37.29 | 87.40 |
| MLP | 64.71 | 0.74 | 46.80 | 69.75 |
| TabNet | 48.68 | 0.85 | 34.58 | 81.51 |

Abbreviations: R^2^, coefficient of determination; RMSE, root mean square error; MAE, mean absolute error; XGBoost, extreme gradient boosting; LightGBM, light gradient boosting machine; CatBoost, categorical boosting; AdaBoost, adaptive boosting; SVM, support vector machine; MLP, multilayer perceptron.

**Table S6.** Statistical description of demographic and clinical characteristics in the training set and test set

| Feature | Train (N=476) | Test (N=119) | Statistics | *P*_value |
| --- | --- | --- | --- | --- |
| daily dose |  |  | W=30390.5 | 0.216 |
| Median (IQR) | 200.00 (150.00–300.00) | 200.00 (120.00–320.00) |  |  |
| Missing | 0 (0) | 0 (0) |  |  |
| weight |  |  | W=30013.0 | 0.313 |
| Median (IQR) | 19.90 (14.00–26.20) | 18.50 (11.80–26.00) |  |  |
| Missing | 0 (0) | 0 (0) |  |  |
| age |  |  | W=29073.0 | 0.654 |
| Median (IQR) | 6.00 (3.00–9.00) | 5.00 (2.00–9.50) |  |  |
| Missing | 0 (0) | 0 (0) |  |  |
| age stratification |  |  | X^2^=0.261 | 0.609 |
| 1 ~ 14 years, median (IQR) | 115 (96.64%) | 455 (95.59%) |  |  |
| ≤1 years, median (IQR) | 4 (3.36%) | 21 (4.41%) |  |  |
| Missing | 0 (0) | 0 (0) |  |  |
| height |  |  | W=29486.0 | 0.488 |
| Median (IQR) | 115.00 (96.00–132.00) | 110.00 (88.00–132.00) |  |  |
| Missing | 0 (0) | 0 (0) |  |  |
| TDM |  |  | W=29346.5 | 0.542 |
| Median (IQR) | 8.55 (5.22–12.86) | 8.53 (4.92–11.98) |  |  |
| Missing | 0 (0) | 0 (0) |  |  |
| CK-MB |  |  | W=28909.5 | 0.726 |
| Median (IQR) | 15.95 (12.00–23.02) | 15.00 (12.00–21.73) |  |  |
| Missing | 0 (0) | 0 (0) |  |  |
| TP |  |  | W=27829.0 | 0.769 |
| Median (IQR) | 63.00 (58.10–67.93) | 62.60 (58.25–68.90) |  |  |
| Missing | 0 (0) | 0 (0) |  |  |
| GLU |  |  | W=27830.0 | 0.769 |
| Median (IQR) | 5.20 (4.54–5.95) | 5.31 (4.62–5.95) |  |  |
| Missing | 0 (0) | 0 (0) |  |  |
| Upper respiratory infection |  |  | χ²=0.392 | 0.531 |
| Infection | 146 (30.67%) | 33 (27.73%) |  |  |
| No infection | 330 (69.33%) | 86 (72.27%) |  |  |
| Missing | 0 (0.0%) | 0 (0.0%) |  |  |
| Imipenem |  |  | χ²=0.827 | 0.363 |
| Use | 119 (25.00%) | 25 (21.01%) |  |  |
| No use | 357 (75.00%) | 94 (78.99%) |  |  |
| Missing | 0 (0.0%) | 0 (0.0%) |  |  |
| Meropenem |  |  | χ²=0.028 | 0.868 |
| Use | 196 (41.18%) | 50 (42.02%) |  |  |
| No use | 280 (58.82%) | 69 (57.98%) |  |  |
| Missing | 0 (0.0%) | 0 (0.0%) |  |  |

Abbreviations: TDM, therapeutic drug monitoring; TP, total protein; GLU, glucose; CK-MB, creatine kinase isoenzyme-MB.

**Table S7.** Mean value of the mask feature based on the TabNet model

| Feature | Step 0 | Step 1 | Step 2 | Step 3 | Average |
| --- | --- | --- | --- | --- | --- |
| weight | 0.095 | 0.298 | 0.06 | 0.371 | 0.206 |
| height | 0.263 | 0.108 | 0.078 | 0.233 | 0.170 |
| age | 0.269 | 0.134 | 0.01 | 0.007 | 0.105 |
| TDM | 0.053 | 0.012 | 0.177 | 0.242 | 0.121 |
| GLU | 0.053 | 0.036 | 0.076 | 0.002 | 0.042 |
| CK-MB | 0.003 | 0.015 | 0.000 | 0.001 | 0.005 |
| TP | 0.144 | 0.074 | 0.076 | 0.005 | 0.075 |
| Imipenem | 0.000 | 0.200 | 0.474 | 0.056 | 0.182 |
| Meropenem | 0.07 | 0.118 | 0.004 | 0.002 | 0.048 |
| upper-respiratory-infection | 0.052 | 0.004 | 0.045 | 0.081 | 0.046 |

Note: Each feature value for each step in the table represents the average of the 119 examples in that step, with the average value for each feature represented in the average column. Abbreviations: TDM, therapeutic drug monitoring; TP, total protein; GLU, glucose; CK-MB, creatine kinase isoenzyme-MB.

**Supplementary Figures**

**
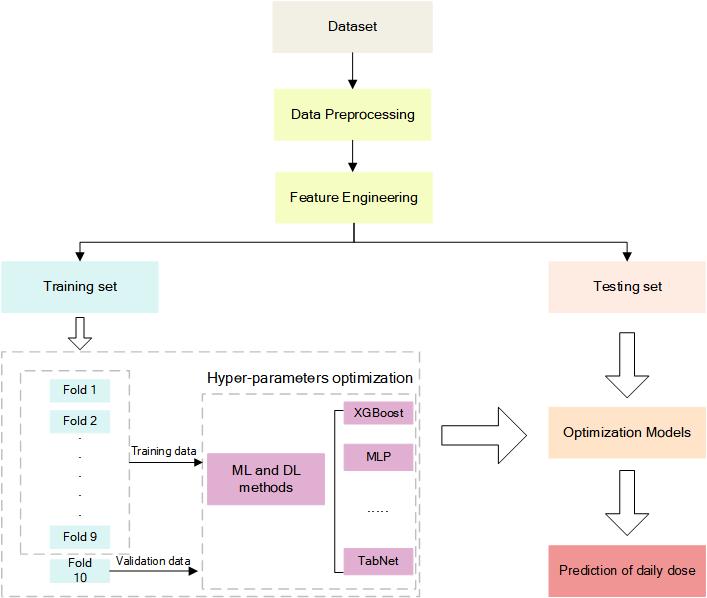
**

**Supplemental Figure S1. Analysis flow diagram.**

**
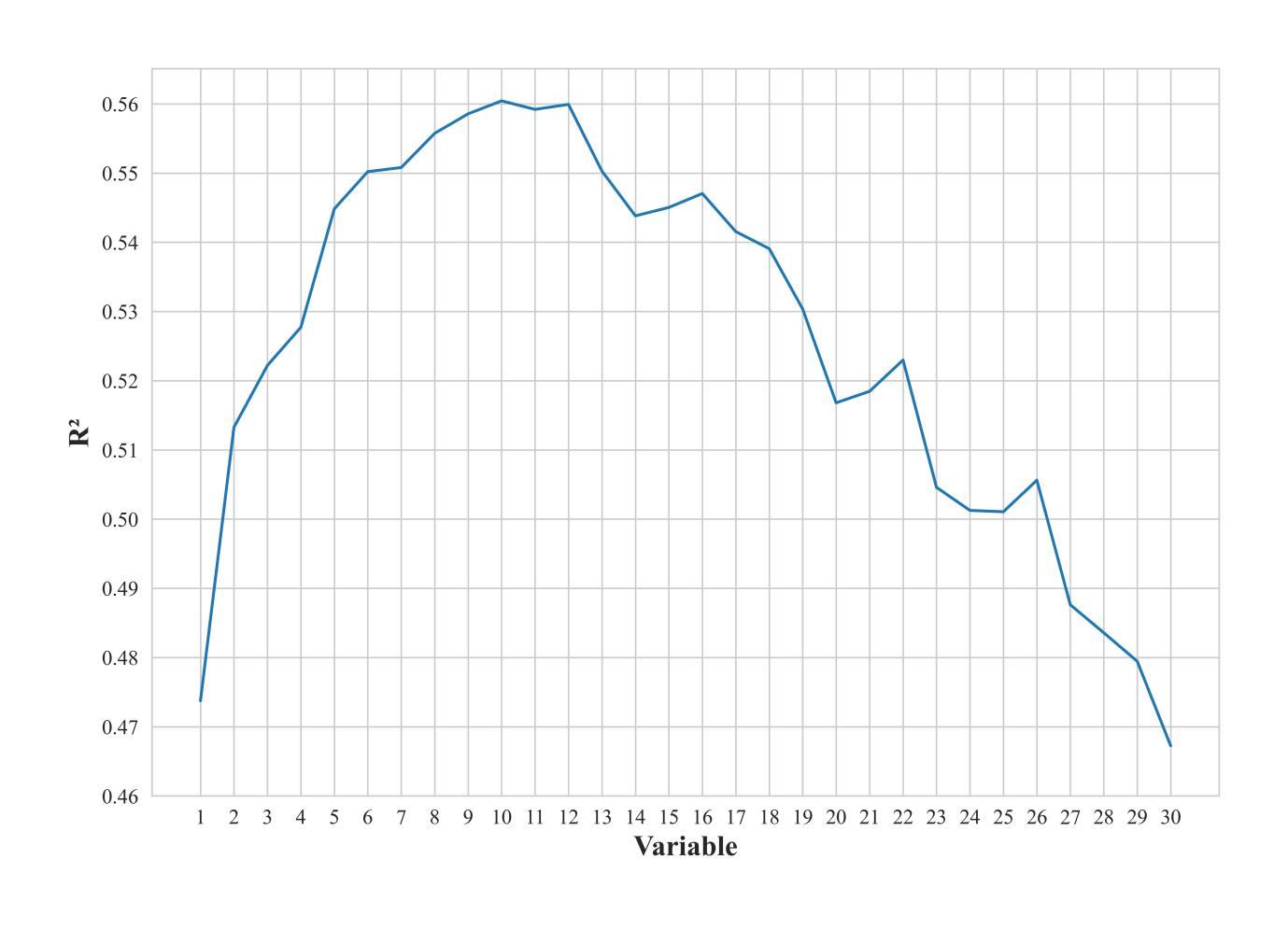
**

**Supplemental Figure S2. Sequential forward feature selection for optimizing predictors of teicoplanin daily dose**

The change in model performance (R^2^) as a candidate variable was sequentially added using an XGBoost-based forward selection approach. Optimal model performance was achieved using ten predictors.

**
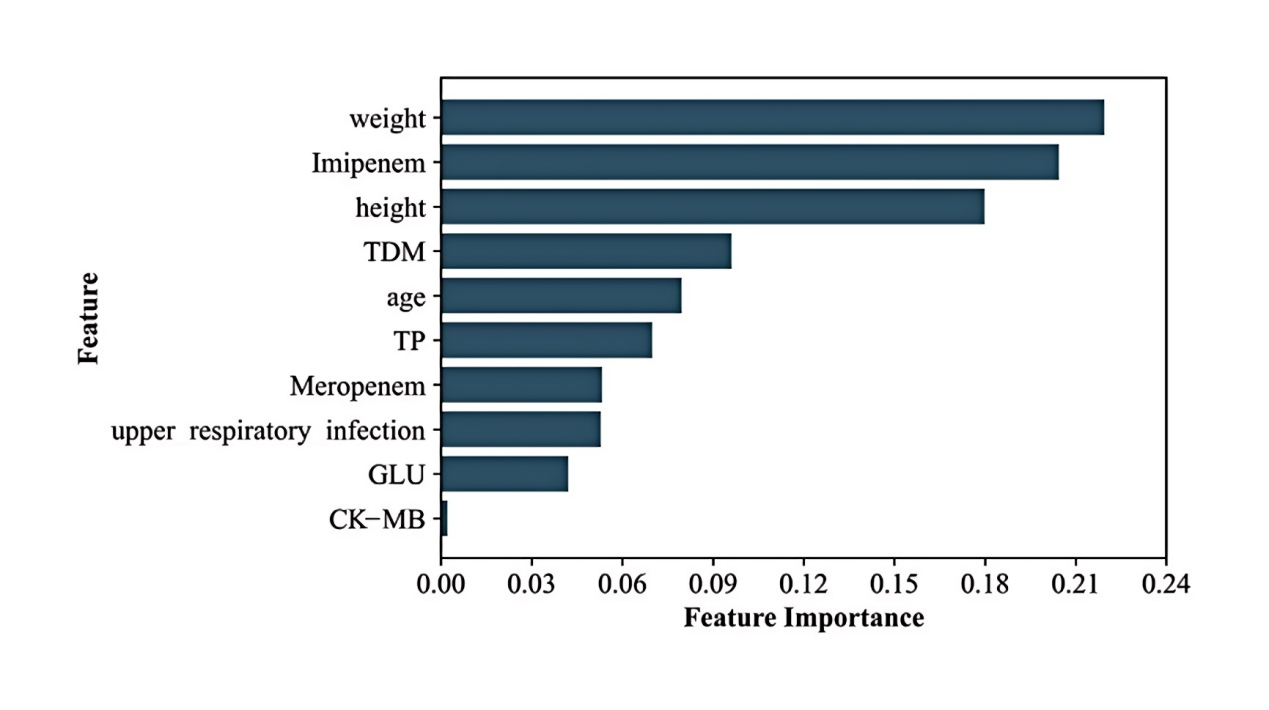
**

**Supplemental Figure S3. A variable importance plot based on the TabNet model.**

Abbreviations: TDM, therapeutic drug monitoring; TP, total protein; GLU, glucose; CK-MB, creatine kinase isoenzyme-MB.

**
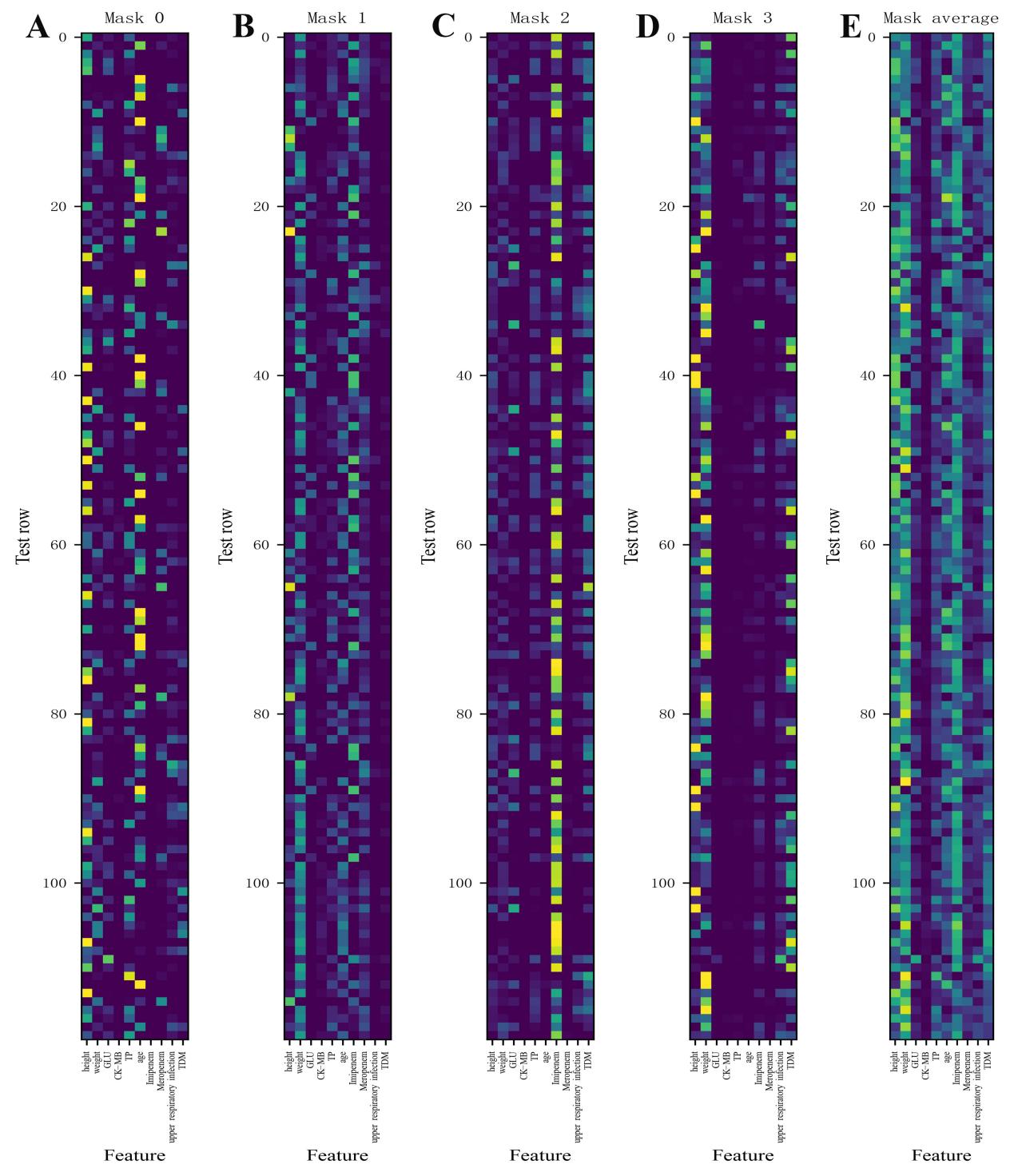
**

**Supplemental Figure S4. The model is based on the feature mask graph of the test set.**

Note: A) to D) represent the feature Mask maps for steps 1 to 4, and E) is the average of the four steps.

Abbreviations: TDM, therapeutic drug monitoring; TP, total protein; GLU, glucose; CK-MB, creatine kinase isoenzyme-MB.


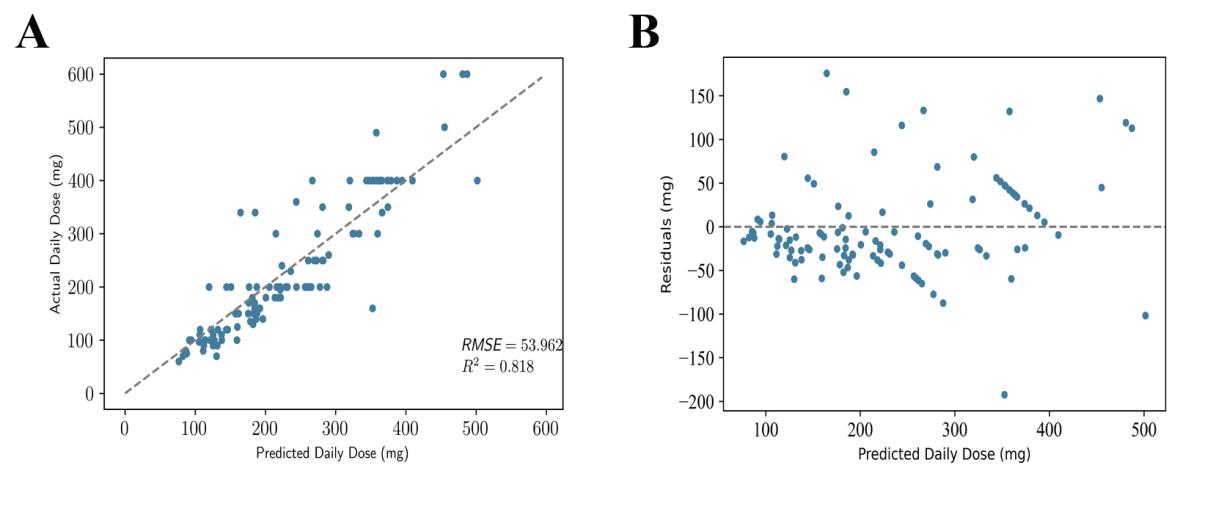


**Supplemental Figure S5. Scatter plot and residual plot based on the test set of the TabNet model in a scholarly context.**

Note: A) and B) show the scatter plot and residual plot of the predicted teicoplanin daily dose and the actual teicoplanin daily dose based on the TabNet model.


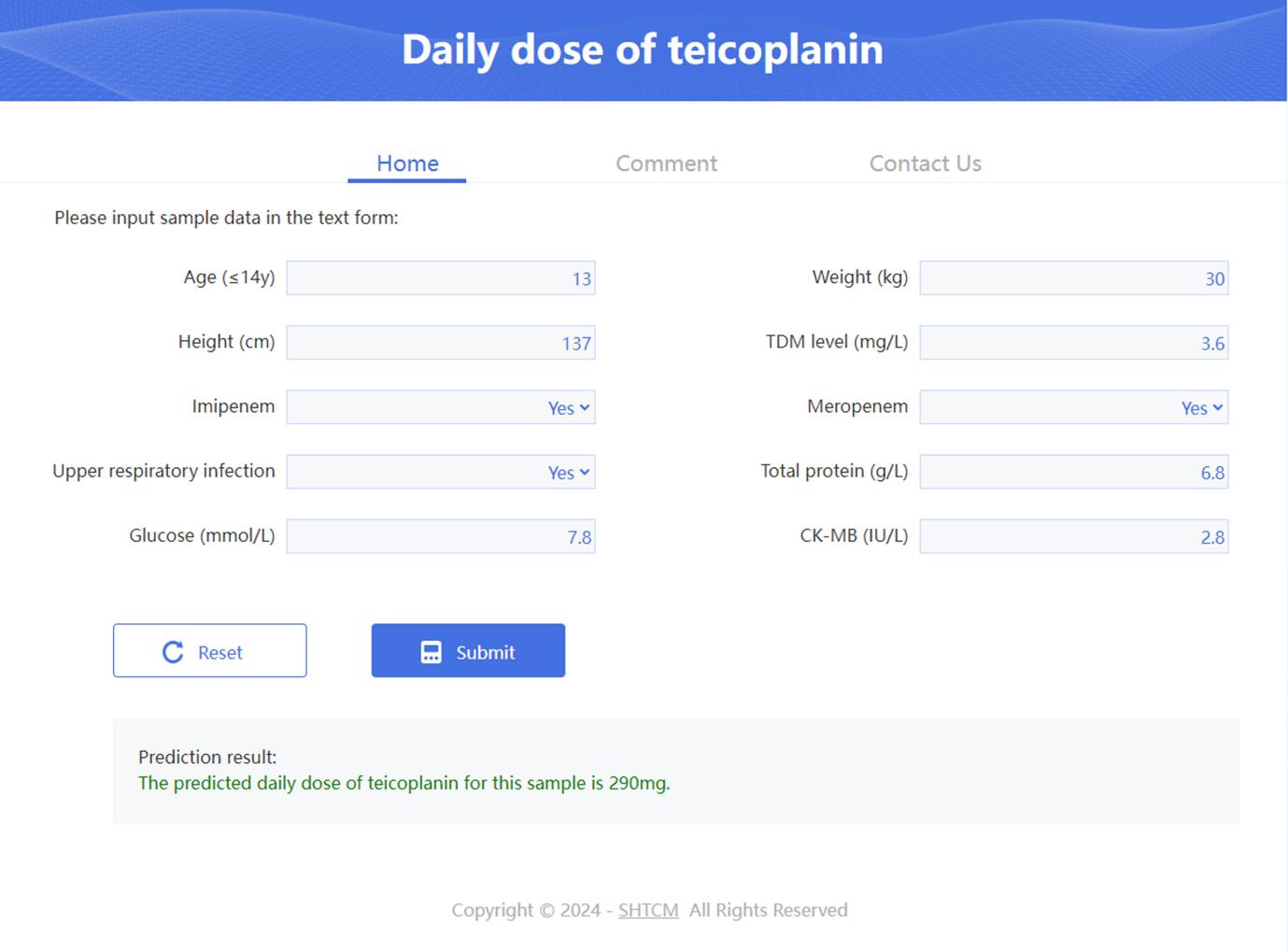


**Supplemental Figure S6**. Online TabNet-based clinical decision-support tool for predicting teicoplanin daily dose (accessible at: https://tdm.ipharma-mtm.com/work/pec5.html)

A screenshot of the web-based interface allows the input of patient-specific clinical and laboratory variables to generate a predicted teicoplanin daily dose consistent with real-world, TDM-guided dosing practice.
